# Parental and professional perspectives on prenatal psychosocial experiences perceived to be linked to autism spectrum disorder: a qualitative content analysis study

**DOI:** 10.1186/s12888-026-07872-x

**Published:** 2026-02-06

**Authors:** Mohammad Hassan Lotfi, Tahmineh Farajkhoda, Fatemeh Doost-Mohammadi, Ali Dehghani, Hossein Fallahzadeh, Tahereh Sadeghiyeh

**Affiliations:** 1 Center for Healthcare Data Modeling, Departments of Biostatistics and Epidemiology, School of Public Health, Shahid Sadoughi University of Medical Sciences, Yazd, Iran; 2 Research Center for Nursing and Midwifery Care, Comprehensive Research Institute for Maternal and Child Health, Department of Midwifery, School of Nursing and Midwifery, Shahid Sadoughi University of Medical Sciences, Yazd, Iran; 3 Clinical Research Development Unit, Shahid Bahonar Hospital, Kerman University of Medical Sciences, Kerman, Iran; 4 Research Center for Addiction and Behavioral Sciences, Department of Psychiatry, School of Medicine, Shahid Sadoughi Hospital, Shahid Sadoughi University of Medical Sciences, Yazd, Iran

**Keywords:** Autism spectrum disorder, Prenatal, Psychosocial, Risk factors, Qualitative, Maternal mental health, Cultural beliefs

## Abstract

**Background:**

While biological contributors to autism spectrum disorder (ASD) are well-established, psychosocial experiences during pregnancy have increasingly been discussed as factors perceived to be associated with ASD. This study explored parents’ and professionals’ perceptions of prenatal psychosocial experiences they believed to be linked to ASD.

**Methods:**

A qualitative content analysis was performed with 22 participants, including 12 parents of children diagnosed with ASD and 10 professionals involved in autism diagnosis and care in Yazd, Iran. Data were collected through semi-structured interviews and analyzed using Graneheim and Lundman’s approach, supported by MAXQDA18. The coding was conducted independently by two researchers. Trustworthiness was maintained using Lincoln and Guba’s criteria. The study adhered to the COREQ 32-item checklist for qualitative reporting.

**Results:**

The analysis revealed two overarching categories: **(1) Psychological-Spiritual Vulnerabilities**, including inadequate emotional well-being during pregnancy, untreated psychological symptoms, and spiritual and metaphysical attributions; and **(2) Sociocultural-Environmental Strains**, encompassing cultural norms and social stigma, interpersonal communication barriers, domestic discord and related conflict, social withdrawal during gestation, economic instability, information**-**related stressors during pregnancy, erroneous health beliefs rooted in limited literacy and Cultural misconceptions about pregnancy treatment choices. Findings demonstrated how internal emotional challenges and external sociocultural stressors were perceived to interact in shaping maternal stress during pregnancy, which participants believed could be associated with subsequent ASD diagnosis.

**Conclusions:**

The study offers a culturally contextualized understanding of the psychosocial experiences that parents and professionals perceive to be associated with a subsequent ASD diagnosis. Integrating culturally sensitive mental health screening and psychosocial support into prenatal care may help address perceived stressors and support maternal well-being, as suggested by participants’ accounts.

**Trial registration:**

Not applicable.

**Clinical trial number:**

Not applicable.

## Background

Autism Spectrum Disorder (ASD) is a neurodevelopmental condition characterized by deficits in communication, social interaction, and behavior patterns [1, 3]. While genetic predisposition contributes to ASD, accumulating research highlights that environmental experiences during the prenatal period are considered influential [4–7].

Among these environmental influences, psychosocial experiences—such as maternal mental health conditions—have gained attention in recent research [8, 9]. Psychosocial distress during pregnancy has been reported to be challenging for maternal well-being and is perceived to relate to neurodevelopmental outcomes, including ASD potentially. Mothers of children with ASD often report experiencing psychological stress, anxiety, or social hardship during pregnancy [10, 11].

Despite the growing body of quantitative evidence, fewer studies have qualitatively explored the subjective experiences of psychosocial stress among women during pregnancy and their perceived connection to ASD. Existing qualitative research suggests that maternal experiences are deeply shaped by cultural, social, and psychological dynamics [12, 13]. However, these studies have not fully captured the diversity of psychosocial experiences, particularly in non-Western or Middle Eastern societies.

Cultural norms and beliefs in different societies may influence how women interpret and respond to psychological distress during pregnancy. For instance, religious interpretations, stigma surrounding mental health, and familial expectations can all affect coping mechanisms, healthcare-seeking behaviors, and subsequent child health outcomes [13, 14]. This cultural lens seems to remain underexplored in the context of ASD.

To address this gap, the present study aims to explore the prenatal psychosocial experiences perceived as relevant to ASD development from the perspectives of parents and professionals in Iran. By examining both maternal-paternal narratives and expert insights, this research seeks to contribute a more comprehensive and culturally grounded understanding of the psychosocial dimensions that participants perceive as related to ASD. These findings may inform more effective and targeted preventive strategies in maternal and child health.

## Methods

### Design and setting

This qualitative study used a conventional content analysis method to explore prenatal psychosocial experiences perceived as relevant to ASD development. The study was conducted in Yazd, Iran, from 2023 to 2024, as part of a broader, sequential, exploratory, mixed-methods research project. This qualitative phase serves as the foundation for a subsequent quantitative study currently underway, which aims to assess the identified perceived associations empirically. Ethical approval was obtained from the Ethics Committee of Shahid Sadoughi University of Medical Sciences, Yazd, Iran (IR.SSU.SPH.REC.1400.227).

### Participants and sampling strategy

A total of 22 participants were recruited: 12 parents (9 mothers and 3 fathers) of children diagnosed with ASD and 10 professionals involved in autism diagnosis and care. ASD diagnosis in children was confirmed using the Gilliam Autism Rating Scale – Second Edition (GARS-2) [15] by a board-certified child psychiatrist.

Parent participants were recruited through convenience sampling from the *Tolou-e-Mehr* and *Baqa* Autism Centers in Yazd, the only officially recognized diagnostic and rehabilitation centers under the Welfare Organization’s supervision at the time of data collection. These centers served as exclusive referral hubs for autism in the region, enhancing the contextual representativeness of the sample.

Out of 30 parents contacted, 12 agreed to participate, resulting in a response rate of 40%. Among those who declined (*n* = 18), documented reasons included work constraints (*n* = 10) and reluctance to discuss sensitive prenatal experiences (*n* = 8).

Participating fathers provided perceived and observational accounts of their wives’ psychosocial states during pregnancy, offering valuable triangulation of family dynamics [16], though potentially subject to recall bias, which was mitigated by cross-checking with maternal prenatal records.

Professional participants were selected through purposive and snowball sampling, starting with the Deputy Director of Rehabilitation at the General Welfare Organization of Yazd, who referred the team to additional qualified informants. This process continued iteratively until data saturation was achieved. Out of 12 professionals contacted, 10 agreed to participate (response rate 83%). Reasons for refusal included scheduling conflicts (*n* = 2).

Maximum variation sampling was applied to ensure diversity in age, gender, and socioeconomic background. Sampling decisions adhered to qualitative research standards based on the principle of data saturation [17, 18]. Operationally, data saturation was monitored continuously during the iterative coding process. New codes and subcategories ceased to emerge after the 18th interview, and this informational redundancy persisted across subsequent interviews, confirming saturation for both parent and professional groups.

### Inclusion and exclusion criteria

Participants were eligible if they were fluent in Persian and willing to share personal or clinical experiences related to psychosocial conditions during pregnancy. Exclusion criteria included current psychiatric hospitalization, cognitive impairment, or refusal to provide written informed consent.

### Data collection procedure

Semi-structured, face-to-face in-depth interviews were conducted individually in private consultation rooms at the study Autism Centers. Each session lasted approximately 40–60 min and began with demographic questions, followed by narrative exploration guided by a semi-structured interview guide.

### Interview guide development

Qualitative Semi-structured interviews were selected to capture nuanced, context-specific perspectives, providing depth but possibly being influenced by subjectivity more than structured questionnaires [19, 20]. To create the interview guide, the multidisciplinary research team, which included experts such as a child and adolescent psychiatrist, collaborated closely. They drew on existing literature about psychosocial experiences perceived to be linked to ASD [8, 19, 20].

The guide featured open-ended questions along with targeted probes designed to explore core areas such as perceived maternal stress, emotional suppression, social or family conflict, and spiritual coping. Through multiple rounds of discussion and expert consultation, the guide was iteratively refined to ensure cultural appropriateness and conceptual depth.

**Sample Questions** *for Parents*:

- “Can you describe your emotional and mental state during pregnancy?”
- “Did any significant life events occur during that time?”
- “Why do you think or perceive your child developed autism?”

*For professionals*:

- “What psychosocial factors during your pregnancy do you believe may be linked to autism in your child?”
- “Are there culturally specific beliefs or behaviors you’ve observed?”

### Supplementary Data

All interviews were audio-recorded with participants’ informed consent and supplemented with concurrent field notes [21] that captured nonverbal cues such as facial expressions, tone, gestures, and emotional intensity. These supplementary observations contributed to data triangulation, enhancing the credibility and contextual depth of the findings.

### Data analysis

Interviews were transcribed verbatim and analyzed using Graneheim and Lundman’s qualitative content analysis framework. MAXQDA18 software facilitated data management. Analysis involved open coding, categorization, and abstraction of both manifest (explicit ) and latent (implicit) content. Coding continued iteratively until data saturation [22, 23]. A combined analytic approach was used to integrate the perspectives of both parents and professionals, enabling the identification of overarching categories that reflected both shared and distinct perspectives.

**Trustworthiness** To ensure rigor, Lincoln and Guba’s four trustworthiness criteria were systematically applied [24]:

- **Credibility** was enhanced through member checking, prolonged engagement, and peer debriefing with experienced qualitative researchers.
- **Dependability** was ensured via transparent documentation, including detailed audit trails and reflexive memoing. Two researchers independently coded the transcripts using a shared framework. Initial inter-coder agreement was calculated at a **minimum of 75%**, representing pre-consensus concordance. Inter-coder reliability was assessed to ensure consistency in data interpretation. Agreement was calculated using simple percentage agreement across different coding levels, including open codes, subcategories, and overarching categories [25]. Reliability was monitored throughout the analysis process and showed slight variation across coding levels (open codes: 75–80%, subcategories: 78–83%, categories: 80–85%).
  Discrepancies between the two primary coders were initially discussed in reflective sessions to reach consensus. When disagreements persisted, a third qualitative expert was consulted. The third-party expert was involved in four out of twenty-two interviews (18%) where divergence remained after the initial reconciliation phase. This systematic approach enhanced the dependability and credibility of the analytic process.
- **Confirmability** was ensured through collaborative coding and the use of maximum variation sampling to reduce individual bias.
- **Transferability** was supported through thick description, illustrative quotations, and adherence to the COREQ 32-item checklist [26].

### Data protection

All data were securely stored on encrypted devices, and participant confidentiality was maintained throughout.

#### Trial registration

As this was a qualitative interview-based study with no interventional component, trial registration was not applicable.

## Results

Participant Characteristics and Coding Summary Data were collected through semi-structured interviews conducted in two groups: (1) 12 parents (9 mothers, 3 fathers) of children diagnosed with ASD, aged between 31 and 42 years (mean ± SD = 35.42 ± 4.63), whose children (aged 3–6 years) were registered at the General Well-being Office of Yazd Province following diagnosis by a pediatric psychiatrist using the Gilliam Autism Rating Scale – Second Edition (GARS-2) [15]; and (2) 10 professionals (4 men, 6 women) aged 25–52 years (mean ± SD = 38.91 ± 3.10), recruited through purposive and snowball sampling techniques. The demographic profiles of parents and their children with ASD, as well as those of participating professionals, are shown in Tables 1 and 2, respectively.

**Table 1:** Demographic characteristics of parent participants and their children diagnosed with autism spectrum disorder

Variable	Categories	frequency
Gender	Males	3
	Females	9
Occupational status	Housekeeper	7
	Employed	4
	Retired	1
Education level	Diploma	2
	BS	8
	MSc	1
	PhD	1
Child’s autism spectrum disorder	Mild	3
	Moderate	7
	Severe	2
Child’s gender	Girl	2
	Boy	10
Parent’s age	35.42 ± 4.63 (Mean ± SD)	

**Table 2:** Demographic profile of professionals and experts participating in the study

Variable	Categories	Frequency
Gender	Males	4
	Females	6
Work experience with ASD	3–15 years	
Occupation	Family and Learning Disorders Counselor	1
	Psychologist and ASD assessor	2
	Pediatric Psychiatrist	1
	Occupational Therapist and Director of a physical rehabilitation school for children with autism	1
	Head of the General Welfare Organization of Yazd Province	1
	Deputy Director of Rehabilitation of the General Welfare Organization of Yazd Province	1
	Director and Founder of the Autism Center	2
	Technical Officer of the Autism Center	1
Education	BS	1
	MSc	4
	Clinical specialists	3
	Ph.D.	2
Age	38.91 ± 3.10 (Mean ± SD)	

Data collection and analysis occurred concurrently using Graneheim and Lundman’s eight-step qualitative content analysis framework. A total of 55 primary codes, 11 subcategories, and 2 main categories emerged from the analysis of combined parental and expert interviews.

Summary of Core Categories The findings represent a triangulated exploration of psychosocial stressors experienced during pregnancy and their perceived link to autism spectrum disorder (ASD), integrating both personal narratives and expert insights. Two major categories, Psychological-Spiritual Vulnerabilities and Sociocultural-Environmental Strains, reflect the complex emotional, relational, and cultural challenges reported by participants. All identified psychological and social stressors were explicitly situated within the prenatal phase, emphasizing the significance of maternal mental and emotional experiences as described by participants. Table 3 presents a summary of the main categories, subcategories, and codes derived from the data analysis.

**Table 3:** Summary of main categories, subcategories, and codes extracted from qualitative interviews with parent and professional participants

Main Category	Subcategory	Sample Codes
Psychological-Spiritual Vulnerabilities	Inadequate emotional well-being during pregnancy	Emotional numbness, hidden distress, unexpressed anxiety, fear of judgment
	Untreated psychological symptoms	Depressive moods, obsessive thoughts, irritability, chronic anxiety
	Spiritual and metaphysical attributions	Divine punishment, ancestral blame, and supernatural beliefs, Parental curse belief
Sociocultural-Environmental Strains	Cultural norms and social stigma	Expectation of maternal silence, fear of emotional expression, stigma around weakness
	Interpersonal communication barriers	Inability to express emotional needs, communication avoidance, and lack of emotional literacy
	Domestic discord and relational conflict	Marital tension, family disputes, emotional pressure during pregnancy, and family conflict
	Social withdrawal during gestation	Isolation at home, avoiding social events, self-imposed retreat
	Economic instability	Job loss during pregnancy, financial worry, and reduced access to care.
	Information-related stressors during pregnancy	Excessive online searching, contradictory information, confusion, and fear, Misinformation from forums, anxiety from conflicting sources, and internet-induced worry
	Erroneous health beliefs rooted in limited literacy	Belief in inactivity, reliance on myths, and unverified prenatal health practices
	Cultural misconceptions about pregnancy treatment choices	Distrust of modern medicine, Preference for traditional or herbal treatments, perceived harm of chemical medications, Cultural beliefs influencing health behaviors, Cultural misconceptions discouraging medical compliance during pregnancy

1. **Psychological-Spiritual Vulnerabilities**: This category encompasses individual psychological experiences and existential interpretations voiced by participants.
  *Inadequate emotional well-being during pregnancy* (reported by 8 parents and 6 professionals): Parents recalled periods of emotional numbness, perceived chronic anxiety, and internalized distress that were often hidden from others. Professionals observed that cultural expectations frequently discourage emotional openness during pregnancy. “I often cried alone during pregnancy but didn’t want anyone to know,” stated one mother (Parent A-2). “I had this constant fear that something was wrong with my baby, but I kept silent because everyone expected me to be happy during pregnancy,” added another (Parent A-5).
  *Untreated psychological symptoms* (7 professionals and 5 parents): Recurrent symptoms such as depressive moods, obsessive tendencies, and irritability were commonly described. Experts highlighted that these perceived and experienced mental health issues, when left unaddressed, may escalate maternal stress and indirectly influence fetal neurodevelopment. “Some mothers internalize stress so deeply it turns into chronic anxiety,” explained one expert (Expert B-1).“I used to get angry very easily during pregnancy, but I didn’t know it was related to stress. I thought it was just hormones,” said a mother (Parent A-8). “I was overwhelmed with worry during pregnancy, fearing something would harm my baby” (Parent A-3).
  *Spiritual and metaphysical beliefs (4 parents and 1 professional): Several participants—*Several participants perceived their child’s autism diagnosis through a spiritual or metaphysical lens, often linking it to events or behaviors during pregnancy. These beliefs included ideas of divine punishment, ancestral transgression, supernatural interference, and being cursed by one’s parents. While some participants reported emotional comfort from these beliefs, professionals noted that families sometimes delayed accessing psychological or medical support.
    “During my pregnancy, I thought I was being punished for being rebellious in my youth,” reflected one mother (Parent A-1). “My mother-in-law said our family’s misfortunes had cursed the baby before birth,” said another parent (Parent A-5).
  Within this overarching subcategory, four distinct subtypes of spiritual interpretation were identified:
  - Religious Misconceptions Hindering Medical Care
    Some families, trusting in divine protection, reported avoiding medical advice. “My husband said God and the Imams will protect us—so I stopped going to clinics” (Parent A-6).
  - Religious Fatalism and Medical Avoidance
    Participants described passive acceptance rather than proactive care-seeking. “Seeing my wife’s struggles as a divine test delayed us from seeking help,” explained a father (Parent A-9). “When mothers attribute pregnancy complications to divine punishment or ancestral sin, they may become less likely to adhere to medical advice, believing that the outcome is already predetermined,” explained one clinical professional (Expert B-7).
  - Magical and Superstitious Thinking
    A smaller group of participants perceived supernatural influences such as black magic, curses, or the evil eye as contributing to prenatal anxiety, leading to feelings of self-blame and helplessness. “I thought I had been cursed and that’s why I was so anxious,” noted a mother (Parent A-8).
  - Positive Religious Coping
    Participants described religious faith as a perceived coping mechanism, helping them manage stress and find emotional grounding during pregnancy. “I talked to God a lot when I was anxious—it helped me calm down,” shared one mother (Parent A-2).
    Professionals emphasized that while spiritual beliefs may aid perceived coping, they should not prevent families from seeking mental health services.
2. **Sociocultural-Environmental Strains**: This category describes social dynamics and broader systemic pressures perceived to contribute to prenatal stress.
  *Cultural norms and social stigma* (6 professionals and 4 parents): Participants reported societal expectations to endure hardship silently, which deterred emotional expression. “Pregnant women in our society are expected to suffer quietly,” shared one expert (Expert B-3). “In my family, showing emotions was seen as a weakness. I kept everything to myself,” admitted one mother (Parent A-6).
  *Interpersonal communication barriers* (5 parents and 4 professionals): Participants described perceived difficulty in expressing emotional needs or initiating dialogue with family members. “It was hard to talk to my husband about how I felt. I didn’t want to be seen as weak,” shared one mother (Parent A-2). “Many mothers are not trained to express psychological needs clearly. This makes early intervention difficult,” noted one expert (Expert B-4).
  *Domestic discord and relational conflict* (7 parents and 6 professionals): Several participants described marital tensions as a source of perceived maternal stress. “My husband and I were fighting constantly during my pregnancy. I was under too much pressure,” recalled one participant (Parent A-5). Also, one father said: “I was under pressure too, but I didn’t know how to support my wife emotionally. She argued a lot without knowing how to stop” (Parent A-12).
     “We often see marital tensions that go unaddressed and escalate maternal anxiety,” said a professional (Expert B-1).
  *Social withdrawal during gestation* (6 parents and 5 professionals): Several mothers described retreating from social activities during pregnancy. “I just stayed in my room most days. I didn’t want to meet anyone or explain how I felt,” said a mother (Parent A-8).“Social isolation due to stigma and lack of understanding can weaken a pregnant woman’s emotional support system,” remarked one professional (Expert B-7).
  *Economic instability* (7 professionals and 6 parents): Although ASD was reported across all income levels, participants described experiencing financial difficulties during pregnancy as a source of perceived stress and as a factor that sometimes shaped how they engaged with prenatal care.
  “When I lost my job during my wife’s pregnancy, she constantly worried about how we’d manage,” shared a father (Parent A-10). “Economic pressure is often invisible but deeply affects how families engage with prenatal care,” noted one expert (Expert B-3).
  *Information-Related Stressors during pregnancy* (6 experts, 3 parents): Participants described how higher parental education, while typically associated with proactive health behaviors, was also perceived to be linked with greater exposure to digital misinformation and information overload during pregnancy. Educated parents reported being highly engaged with online information—using social media platforms (e.g., Instagram, Telegram, WhatsApp), local apps (e.g., Bale, Rubika, Eita, Soroush), online forums, health websites, and even AI-based tools such as ChatGPT—to better understand pregnancy and autism. However, this ongoing information-seeking was often described as contributing to feelings of anxiety and confusion rather than reassurance.
  Participants described several main types of problematic digital engagement:
  - Passive exposure to social media was described as immersing mothers in emotionally charged stories and fear-inducing narratives about autism. Algorithm-driven content loops were perceived to intensify these worries. As one parent shared, *“I kept reading about autism on Instagram*,* and every new thing I read made me more scared during pregnancy”* (Parent A-4). An expert similarly observed that *“educated mothers often search the web excessively and fall into a spiral of worry and misinformation”* (Expert B-5).
  - Active searching through online forums and unverified websites was perceived to deepen feelings of uncertainty, as conflicting claims and pseudoscientific explanations blurred the line between credible and false information. *“I read so many things on forums that contradicted each other—it made me feel helpless”* (Parent A-1).
  - Interactive engagement with AI-based tools (Artificial Intelligence tools) such as ChatGPT (Chat-based Generative Pre-trained Transformer) was described as occasionally creating a perceived sense of reassurance or confusion, depending on how mothers interpreted the responses. One mother explained, *“During my pregnancy*,* I even asked ChatGPT about autism. At first*,* the answers seemed professional*,* but later I found contradictions online—it left me more confused”* (Parent A-7).
    Professionals emphasized that such cognitive and emotional overload appeared particularly salient among educated parents who felt a strong responsibility to “get everything right.” This perceived sense of obligation, combined with the unfiltered digital environment, was interpreted as transforming knowledge-seeking into a psychosocial stressor. As one expert noted, *“Most mothers who worried excessively were well-educated—they read a lot*,* but much of what they found was confusing or* exaggerated” (Expert B-2).
    Collectively, these accounts illustrate how digital engagement—shaped by educational background and the desire for certainty—was perceived to undermine emotional stability during pregnancy. The convergence of information overload, misinformation, and algorithm-driven content was experienced by participants as a distinct psychosocial strain within modern maternal life, reflecting how digital technologies were perceived to amplify both awareness and anxiety.”
    *Erroneous health beliefs and limited literacy* (7 experts): Participants mentioned perceived misconceptions and superstitions that influenced prenatal behavior. “I thought staying completely inactive during pregnancy was good for the baby. I now know that was wrong,” shared a mother (Parent A-11). “Many families base their health decisions on myths or unverified sources, which complicates prenatal care,” emphasized one professional (Expert B-2).
    *Cultural misconceptions about pregnancy treatment choices* (6 parents): Participants described how culturally rooted perceived misconceptions led some to prefer herbal remedies over medically recommended prenatal supplements. This resistance was often shaped by the mother’s perceived beliefs or influenced by the husband, reflecting the complex relationship between cultural norms and maternal perceived autonomy in prenatal decision-making.
      “My husband didn’t let me take the iron pills, saying chemical things hurt the baby—but he made me drink some traditional herbs instead,” said one mother (Parent A-3).
      “In our village, herbal remedies are a trusted part of our tradition. I preferred them over synthetic pills, even though the doctor said they were necessary,” shared Parent A-7.
      Overall, the findings were interpreted as reflecting perceived psychosocial vulnerabilities during pregnancy—shaped by both individual emotional experiences and broader sociocultural dynamics—that participants believed to be relevant in understanding perceived links to ASD. While these insights do not imply direct causality, the integration of parental narratives and professional observations provides a culturally contextualized understanding of how such perceptions may shape maternal well-being during gestation.

## Discussion

This qualitative study explored how parents and professionals in Yazd, Iran, perceive prenatal psychosocial experiences to be associated with autism spectrum disorder (ASD) diagnosis. Using qualitative content analysis based on Graneheim and Lundman’s approach, two primary categories emerged inductively from participants’ narratives: *Psychological-Spiritual Vulnerabilities* and *Sociocultural-Environmental Strains*. To enhance theoretical coherence, these two main categories were subsequently mapped onto a three-level framework—individual, familial, and sociocultural-environmental—inspired by Bronfenbrenner’s ecological systems theory [27]. This post-hoc mapping aligns the inductive findings with interconnected layers of influence (micro, meso, exo, and macrosystem) without altering the primary analysis.

Semi-structured interviews were employed to capture culturally specific, emotionally nuanced insights in Yazd, which standardized questionnaires might overlook due to their rigidity [17]. This methodological choice uncovered unique sociocultural themes, aligning with the study’s aim to explore subjective experiences in a less-studied Iranian context [21, 23]. These interpretive findings complement standardized tools [28, 29], offering context-sensitive insights without aiming to generalize or establish causality.

The inductively derived categories reflect perceived psychosocial stressors, such as prenatal stress, family conflict, and spiritual interpretations, which participants described as potentially linked to ASD. These factors are not exclusive to ASD and may be relevant to other neurodevelopmental conditions, such as Attention Deficit Hyperactivity Disorder (ADHD), Global Developmental Delay (GDD), and Developmental Language Disorder (DLD) [30, 31]. By mapping these onto Bronfenbrenner’s framework, the study highlights how individual (microsystem), familial (mesosystem), and sociocultural-environmental factors (exosystem) were perceived to interact in shaping maternal experiences related to ASD, within a culturally specific context.

### Individual level (psychological-spiritual vulnerabilities)

A prominent perspective was psychological distress as perceived by participants during pregnancy, particularly anxiety and depressive moods. This aligns with prior literature reporting associations between perceived prenatal stress and later developmental concerns [32, 33]. For example, O’Donnell et al. (2014) found that maternal mood in pregnancy had long-term implications for childhood psychopathology [33], while Brown et al. (2017) linked prenatal depression to increased ASD risk [34]. However, not all research is in agreement—other studies have shown limited or no direct causality, suggesting that contextual and environmental modifiers play a mediating role.

Some participants described spiritual faith as a source of emotional regulation, while other spiritual beliefs, such as reliance on divine protection or superstitions, were perceived to delay professional care-seeking. For example, certain mothers reported refraining from seeking psychological support due to reliance on prayers or spiritual remedies. This dual role underscores the need for culturally sensitive prenatal education that respects beliefs while promoting evidence-informed care, as observed in other collectivist societies, a pattern also noted by Ahmed et al. (2021) in Pakistan [35].

### Familial level (family dynamics and domestic stressors)

Inclusion of paternal narratives enriched the findings by providing perceptions of maternal stress, social expectations, and family interactions. Fathers’ observations highlighted how family tensions and communication barriers were perceived to reduce social support and complicate maternal coping during pregnancy, rather than directly causing developmental outcomes.

Health-related decisions were perceived to be influenced by spousal authority, reflecting gendered dynamics that participants believed could constrain maternal autonomy. These accounts emphasize the importance of considering both parental and familial perspectives in understanding perceived psychosocial experiences [36, 37].

### Sociocultural-environmental level

In Yazd, cultural norms emphasizing maternal resilience, emotional restraint, and the preservation of family honor often intensified psychosocial stress during pregnancy. Many women concealed distress to avoid appearing weak or burdening relatives, while some men prioritized spiritual submission over seeking psychological care. These dynamics, reinforced by mental health stigma and traditional gender roles, reflect Mandell and Novak’s (2005) observations on how culturally embedded explanatory models shape perceptions of autism and influence engagement with psychosocial services [38]. Compared with more urbanized Iranian settings such as Tehran, Yazd’s religious conservatism and collectivist values appeared to amplify spiritual attributions for distress and discourage open mental health discussions. This mirrors patterns in other collectivist societies—from East Asia, where family honor limits disclosure, to Sub-Saharan Africa, where spiritual interpretations dominate health-seeking behaviors—highlighting the potential for cross-cultural adaptation of interventions [39–41].

Beyond traditional beliefs, the sociocultural landscape of Yazd is now shaped by pervasive digital influences. Urban and highly educated mothers frequently described experiencing information overload arising from exposure to vast, conflicting, and often inaccurate content online (Mars et al., 2021; Choi et al., 2017; [42, 43]). Consistent with Choi et al.’s findings, a paradox emerged: higher education predicted greater online engagement but also heightened confusion and anxiety. Participants described social media algorithms—particularly on platforms like Instagram—as amplifying emotionally charged or fear-inducing content based on previous engagement. This repetitive exposure created “echo chambers of fear,” in which distressing narratives were continually reinforced, fostering rumination and uncertainty.

Interactions with AI-based tools such as ChatGPT were similarly described as a double-edged experience. While these tools provided rapid access to information, their authoritative tone often lent undue credibility to unverified or contradictory claims. In the absence of professional oversight, such interactions blurred the boundary between expert guidance and misinformation, intensifying emotional confusion. Collectively, these digital design features—algorithmic reinforcement and perceived authority—were perceived as psychosocial stressors contributing to a broader sense of digital overload.

Patterns of exposure also reflected social and educational divides. Passive, algorithm-mediated scrolling commonly led to uncritical absorption of emotionally charged narratives, while active participation in online parenting forums sometimes provided peer support but also reproduced misinformation when users lacked digital health literacy. Conversely, rural mothers—less digitally connected—were more likely to rely on traditional remedies and community-based knowledge, often citing the “natural safety” of herbal teas. Such preferences, supported by spouses and reflecting gendered decision-making, parallel findings in South Asian contexts [40, 41, 44, 45] but diverge from Western cautionary guidelines on herbal use [42].

Taken together, these findings at the sociocultural-environmental level suggest that prenatal psychosocial experiences are influenced not only by traditional cultural norms but also by the modern information ecology in which expectant mothers are embedded. Consequently, interventions aimed at this level might benefit from addressing digital health literacy, critical appraisal skills, and culturally sensitive channels for professional guidance, to help mitigate the psychosocial strain perceived to be linked with information overload, while recognizing that additional factors at the individual and familial levels also contribute to maternal experience.

Despite broad agreement in the literature on the negative impact of psychosocial stress, some studies argue that maternal resilience, protective social networks, and adaptive coping strategies can buffer these effects. This contrast underscores the need for more nuanced exploration into which factors moderate or mediate the relationship between stress and ASD outcomes [19].

Finally, *while our data suggest* that autism spectrum disorder (ASD) is not limited to any particular socioeconomic group, some participants described how disparities in access to information, quality of care, and institutional support may shape how prenatal stress is experienced and managed. These observations align with Lee’s (2021) suggestion that socioeconomic class can influence diagnostic pathways and service utilization, even when the prevalence of neurodevelopmental conditions remains similar across social strata [46].

This study offers a context-sensitive understanding of prenatal psychosocial experiences perceived as potential risks by both parents and professionals within an Iranian setting. While the findings are situated within a specific cultural and geographic context, some of the reported stressors and explanatory models—such as stigma, spiritual attributions, and emotional suppression—may resonate with patterns observed in other non-Western or collectivist societies [47, 48]. Further comparative research is needed to explore how these culturally shaped interpretations vary across contexts and to distinguish shared elements from those that are context-specific.

It should also be emphasized that this qualitative, retrospective study reflects participants’ subjective interpretations and perceived experiences rather than objective or causal relationships between prenatal psychosocial factors and autism spectrum disorder.

### Policy implications

Although exploratory and context-bound, participants’ insights suggest potential strategies for culturally responsive psychosocial screening, mental health support, and digital health literacy interventions, emphasizing perceived stressors rather than established causal links. However, any such efforts should be developed with careful consideration of local beliefs, norms, and existing health system structures.

## Conclusions

This study provides a context-sensitive understanding of how parents and professionals perceive psychosocial experiences during pregnancy as linked to ASD. The findings emphasize the salience of maternal emotional states, familial dynamics, and cultural interpretations in shaping these perceptions. While not aiming to infer causality, the study sheds light on how such perceived factors are subjectively understood within a specific sociocultural context. These findings, based on subjective perceptions, highlight the need for further research to explore the interplay of psychosocial stressors and ASD.

### Limitations

As with many retrospective qualitative studies, this research is inherently subject to recall bias, particularly when addressing emotionally sensitive issues such as psychosocial stress during pregnancy. To reduce this risk, interviews were conducted with parents of children under six, as their relatively recent experiences enhance memory accuracy and improve recall of prenatal events. Additionally, anchored questioning and methodological triangulation were employed to enhance the credibility and depth of the findings.

To further verify participants’ narratives, especially those provided by fathers, self-reported psychosocial experiences were cross-checked with maternal psychiatric and prenatal care records. Given that mental health assessments are routinely integrated into Iran’s national prenatal care system, these records offered a reliable basis for validating key psychological indicators during pregnancy.

A further limitation is the underrepresentation of fathers (3 out of 12 participants), mainly due to scheduling conflicts and work-related absences during recruitment, which limited the comprehensiveness of paternal perspectives on maternal psychosocial stress. This limited paternal representation may have skewed our understanding of key aspects such as domestic discord, interpersonal communication barriers, and cultural misconceptions about treatment choices. A more balanced inclusion of fathers could have provided a richer and more nuanced picture of family dynamics during pregnancy.

Given that the sample was drawn from Yazd and comprised families with access to formal autism diagnostic services, the findings may not fully represent the experiences of underserved or geographically diverse populations. Therefore, caution is advised when generalizing these results to broader cultural or healthcare contexts. Furthermore, the findings represent participants’ personal perceptions and recollections and therefore may not reflect objective or causal associations with ASD.

### Recommendations for future research

Future research should further investigate how culturally specific beliefs, particularly spiritual or metaphysical perspectives, shape perceptions of prenatal risk and influence maternal mental health across diverse sociocultural settings. Given the limited participation of fathers in the present study, employing alternative recruitment strategies (e.g., telephone-based or weekend interviews) may facilitate the inclusion of paternal viewpoints.

Comparative qualitative studies involving parents of children with other developmental conditions (e.g., global developmental delay, developmental language disorder, attention-deficit/hyperactivity disorder) are warranted to determine whether the psychosocial stressors identified are unique to autism spectrum disorder or represent broader parenting challenges. As this was an exploratory study, confirmatory comparative research is necessary to establish the specificity of these perceived factors to ASD.

Furthermore, applied research should develop culturally adapted psychosocial screening tools, like standardized questionnaires for anxiety, depression, and cultural beliefs, for use by community health workers or midwives in Iran’s primary healthcare. Pilot programs, coordinated with the Ministry of Health, should be implemented in Yazd’s rural and urban health centers. Specific strategies could include:

(1) collaborating with local religious leaders to co-design screening tools that incorporate spiritual beliefs; (2) integrating these tools into existing prenatal check-ups via mobile apps for urban areas and community health worker training for rural settings; and (3) evaluating pilot outcomes through mixed-methods feedback to ensure scalability within Iran’s primary healthcare system.

## Data Availability

The datasets generated and analyzed during the current study are not publicly available due to the sensitive nature of qualitative interview content, but are available from the corresponding author on reasonable request.

## Abbreviations

ASD: Autism Spectrum Disorder
GARS-2: Gilliam Autism Rating Scale—Second Edition
ADHD: Attention-deficit hyperactivity disorder
GDD: Global Developmental Delay
DLD: Developmental Language Disorder
AI: Artificial Intelligence
Chat GPT: Chat-based Generative Pre-trained Transformer
